# Misleading Health-Related Information Promoted Through Video-Based Social Media: Anorexia on YouTube

**DOI:** 10.2196/jmir.2237

**Published:** 2013-02-13

**Authors:** Shabbir Syed-Abdul, Luis Fernandez-Luque, Wen-Shan Jian, Yu-Chuan Li, Steven Crain, Min-Huei Hsu, Yao-Chin Wang, Dorjsuren Khandregzen, Enkhzaya Chuluunbaatar, Phung Anh Nguyen, Der-Ming Liou

**Affiliations:** ^1^Graduate Institute of Medical InformaticsCollege of Medical Science and TechnologyTaipei Medical UniversityTaipeiTaiwan; ^2^Institute of Biomedical InformaticsNational Yang Ming UniversityTaipeiTaiwan; ^3^NorutTromsoNorway; ^4^School of Health Care AdministrationTaipei Medical UniversityTaipeiTaiwan; ^5^Wan Fang HospitalDepartment of DermatologyTaipei Medical UniversityTaipeiTaiwan; ^6^School of Computational Science and EngineeringGeorgia Institute of TechnologyAtlanta, GAUnited States; ^7^Department of Computer ScienceOberlin CollegeOberlin, OHUnited States; ^8^Institute of Public Health SciencesNational Yang Ming UniversityTaipeiTaiwan

**Keywords:** Medical Informatics, Internet, Online videos, YouTube, Eating Disorder, Anorexia Nervosa, Social Network

## Abstract

**Introduction:**

The amount of information being uploaded onto social video platforms, such as YouTube, Vimeo, and Veoh, continues to spiral, making it increasingly difficult to discern reliable health information from misleading content. There are thousands of YouTube videos promoting misleading information about anorexia (eg, anorexia as a healthy lifestyle).

**Objective:**

The aim of this study was to investigate anorexia-related misinformation disseminated through YouTube videos.

**Methods:**

We retrieved YouTube videos related to anorexia using the keywords anorexia, anorexia nervosa, proana, and thinspo on October 10, 2011.Three doctors reviewed 140 videos with approximately 11 hours of video content, classifying them as informative, pro-anorexia, or others. By *informative* we mean content describing the health consequences of anorexia and advice on how to recover from it; by *pro-anorexia* we mean videos promoting anorexia as a fashion, a source of beauty, and that share tips and methods for becoming and remaining anorexic. The 40 most-viewed videos (20 informative and 20 pro-anorexia videos) were assessed to gauge viewer behavior.

**Results:**

The interrater agreement of classification was moderate (Fleiss’ kappa=0.5), with 29.3% (n=41) being rated as pro-anorexia, 55.7% (n=78) as informative, and 15.0% (n=21) as others. Pro-anorexia videos were favored 3 times more than informative videos (odds ratio [OR] 3.3, 95% CI 3.3-3.4, *P*<.001).

**Conclusions:**

Pro-anorexia information was identified in 29.3% of anorexia-related videos. Pro-anorexia videos are less common than informative videos; however, in proportional terms, pro-anorexia content is more highly favored and rated by its viewers. Efforts should focus on raising awareness, particularly among teenagers, about the trustworthiness of online information about beauty and healthy lifestyles. Health authorities producing videos to combat anorexia should consider involving celebrities and models to reach a wider audience. More research is needed to study the characteristics of pro-anorexia videos in order to develop algorithms that will automatically detect and filter those videos before they become popular.

## Introduction

Social networking has emerged as a new channel for seeking information, and also for creating and exchanging user-generated content among peers [1]. An increasing amount of content is being disseminated on social video platforms, such as YouTube, Vimeo, and Veoh. For example, approximately 26% of teenagers aged 13 to 17 years create and upload videos [2], and approximately 60 hours’ of content is uploaded onto the video-sharing platform YouTube each minute, with more than 4 billion page views every day [3]. YouTube is gaining popularity among American and European health care providers [4,5] not just as a video repository, but also as a social network where users interact to build trust with comments and favorites [6]. Approximately 100 million people take some form of social action on YouTube (eg, likes, shares, and comments) every week [3].

Many different stakeholders generate health-related content on social media platforms [7]. For example, health consumers publish videos about their diseases on YouTube [8-10], whereas health care professionals collaborate to increase the quality of articles published in Wikipedia [11]. Traditional health portals, such as NHS Choices, Mayo Clinic, and PubMed, use social media channels (eg, YouTube and Facebook) to distribute their content [7], whereas an increasing number of health consumers search for health information on social media channels [12]. However, finding informative and trustworthy online health information is hampered by the vast amount of information available [13-16], the quality of which is heterogeneous. This is clearly the situation faced by users of YouTube [6,17-24] where thousands of videos promote misleading information, such as disparaging vaccinations [17,23,24]. This clearly jeopardizes the safety of social media content.

**Figure 1:**
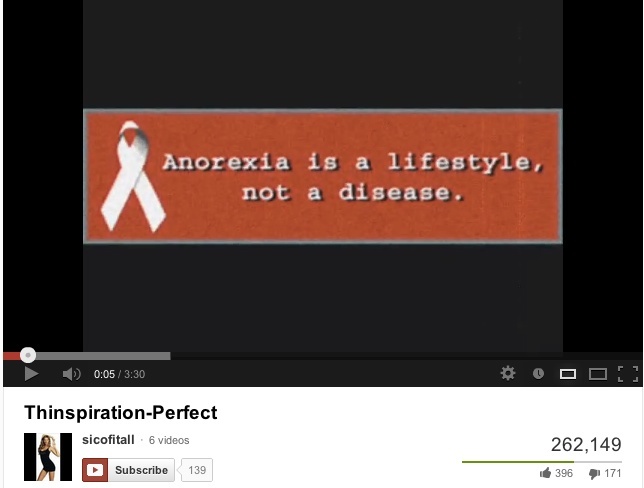
Screenshot of a pro-anorexia video promoting misinformation.

Online information about anorexia provides a good example of potentially harmful online information. Anorexia is an eating disorder which has a huge impact on the health and quality of life of sufferers [25], and these people commonly engage in the creation of online content promoting anorexia as a lifestyle (see Figure 1 and Multimedia Appendix 1) [26-35]. Pro-anorexia websites have been defined as those encouraging disordered eating [26], promoting anorexia as a fashion or as a source of beauty, and sharing tips and tactics on how to become and remain anorexic. However, for people affected by anorexia who deny the disease, pro-anorexia information can be “trustworthy” and very informative. In this paper, we consider pro-anorexia information as misleading because, as we explain subsequently, it can be detrimental to a person’s health [28,32-35]. On the other hand, there are also videos that inform about the health consequences of anorexia (see Multimedia Appendix 2 for an example of an informative video).

The desire to be thin (*thinspiration*) leads adolescents, mainly females, to develop low perceptions of their own body image [34-37], together with unrealistic ideals of thinness based on models depicted on the Internet. Recent studies have identified pro-anorexia and pro-eating-disorder websites as negatively affecting females’ perceptions of their body image [8,32,34]. Many pro-anorexia members share pictures and videos of extremely thin models, reflecting the current media trend toward very thin beauty canons, which is pushing many teenagers toward unhealthy eating habits [35].

Pro-anorexia content has the potential to become a public health concern. One study showed that 13% of 1575 female undergraduates reported viewing 1 or more pro-eating disorder sites, rating them higher on eating measures and body image disturbances [30]. Pro-anorexia content has been found to exacerbate eating disorders and promote anorexic lifestyles [32,34]. In a recent review, Rouleau and von Ranson [32] summarized 3 main reasons why pro-anorexia communities can be harmful: (1) they claim to provide support, (2) they promote disordered eating, and (3) they discourage people from seeking help or trying to recover.

Pro-anorexia content tends to be more popular among young people who are more susceptible to concerns about body weight [36]. Most people affected by anorexia fall within the age group in which Internet and social media are used heavily [25]. Studies suggest that pro-anorexia content is developed within online communities [27,29]. Therefore, one would expect a high presence of pro-anorexia content on social media platforms. Despite the popularity of social media, little is known about pro-anorexia content and the use of social media platforms [33], especially on multimedia platforms, such as videos (ie, YouTube) and images (ie, Flickr). To our knowledge, this study is the first to investigate pro-anorexia-related information disseminated through YouTube videos.

## Methods

**Figure 2:**
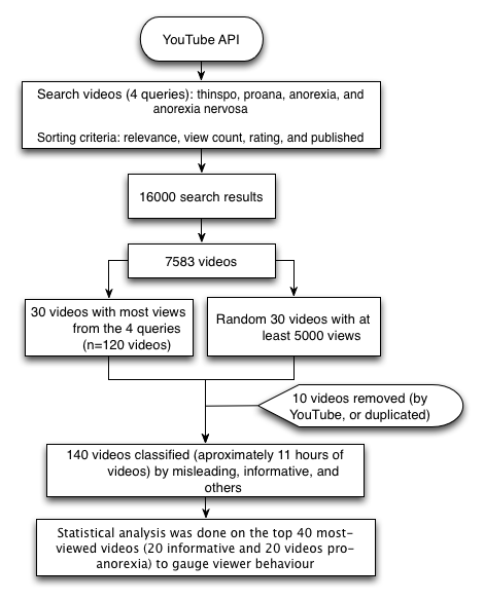
Study design.

As shown in Figure 2, we used the YouTube application programming interface (API) to search for videos with queries related to anorexia using the keywords anorexia, anorexia nervosa, proana, and thinspo (inspiration to become thin) on October 10, 2011. We retrieved up to 4000 results for each query and sorting criteria (relevance, uploaded, number of views, and rating). In total, 16,000 search results were retrieved containing 7583 videos uploaded by 3968 users.

We selected the 30 most-viewed videos for the previously mentioned 4 keywords (n=120) and a subset of 30 random videos with at least 5000 views for classification by experts. Out of the 150 videos selected, only 140 were analyzed because 8 videos were retrieved in several of the queries and 2 videos were removed from YouTube in the middle of the reviewing process. They may have been deleted because of a copyright issue or a violation of YouTube regulations. An additional 21 videos were in a European language (Spanish, Italian, and Portuguese) and experts with knowledge of these languages were contacted for clarification. The 140 videos totaled 11 hours of video.

Three independent physicians (Y-CW, EC, and DK) reviewed the 140 videos. A predetermined classification criterion was agreed upon based on a review of the literature and group discussions among the authors. A subset of videos was used to test the classification criterion, although these videos were not included in the study analysis. Finally, reviewers received a written description for each category. Videos describing anorexia as an eating disorder, explaining the consequences of malnutrition on health, or suggesting how to recover from this condition were rated as informative. In contrast, videos describing anorexia as a fashion, a source of beauty, a healthy lifestyle, included ways of avoiding meals, or included tips on how to become and remain anorexic were rated as pro-anorexia. Finally, videos that were not related to anorexia or eating behavior were rated as others. The interrater agreement was estimated by Fleiss’ kappa. A majority consensus was not reached for 10 videos; therefore, 3 additional reviewers (LFL, SAS, and CS) reclassified these videos by consensus after watching them together.

We selected the top 40 videos (the 20 most-viewed pro-anorexia videos and the 20 most-viewed videos from the informative categories) because we were interested in the videos with the maximum number of viewers, and users normally just browse the first pages of search results. These 40 videos had a total of 61.13 million views, which is a large enough sample size to understand the characteristic features of the viewers. The statistics software SPSS v17 (SPSS Inc, Chicago, IL, USA) was used to analyze the different features of the pro-anorexia and informative videos. We also analyzed the content of videos and viewership whenever demographic information was available.

## Results

Of the 140 videos, 41 (29.3%) were rated as pro-anorexia, 78 (55.7%) as informative, and 21 (15.0%) as others (see Table 1). The interrater agreement of their classification was moderate (Fleiss’ kappa = 0.5) [38]. The random selection of 30 videos with at least 5000 or more views had similar percentages: 10 (33%) were pro-anorexia videos, 17 (57%) were informative, and 3 (10%) were others. If this percentage is extrapolated to the total dataset, we can assume that YouTube contains approximately 2222 pro-anorexia videos (29.3% of 7583).

**Table 1:** Results of the classification of anorexia-related videos on YouTube according to the informative nature of the videos.

Selected YouTube videos	Total, n	Video classification, n (%)		
		Pro-anorexia	Informative	Others
Top 30 videos with most views for each query	110	32 (29.0%)	61 (55.5%)	17 (15.5%)
Random selection of videos with more than 5000 views	30	10 (33%)	17 (57%)	3 (10%)
Total reviewed videos	140	41 (29.3%)	78 (55.7%)	21 (15.0%)

Furthermore, the 40 most-viewed videos (20 pro-anorexia and 20 informative videos) with a total of 61.13 million views were assessed to understand the behavior of the viewers (see Table 2). Users normally just browse the first pages retrieved by search engines, thus making it important to study the top results. Pro-anorexia videos were favored 3 times more than informative videos. The response rate was estimated from the number of viewers who clicked on the like/dislike icon over total views. Pro-anorexia video viewers responded twice as often as those of informative videos.

**Figure 3:**
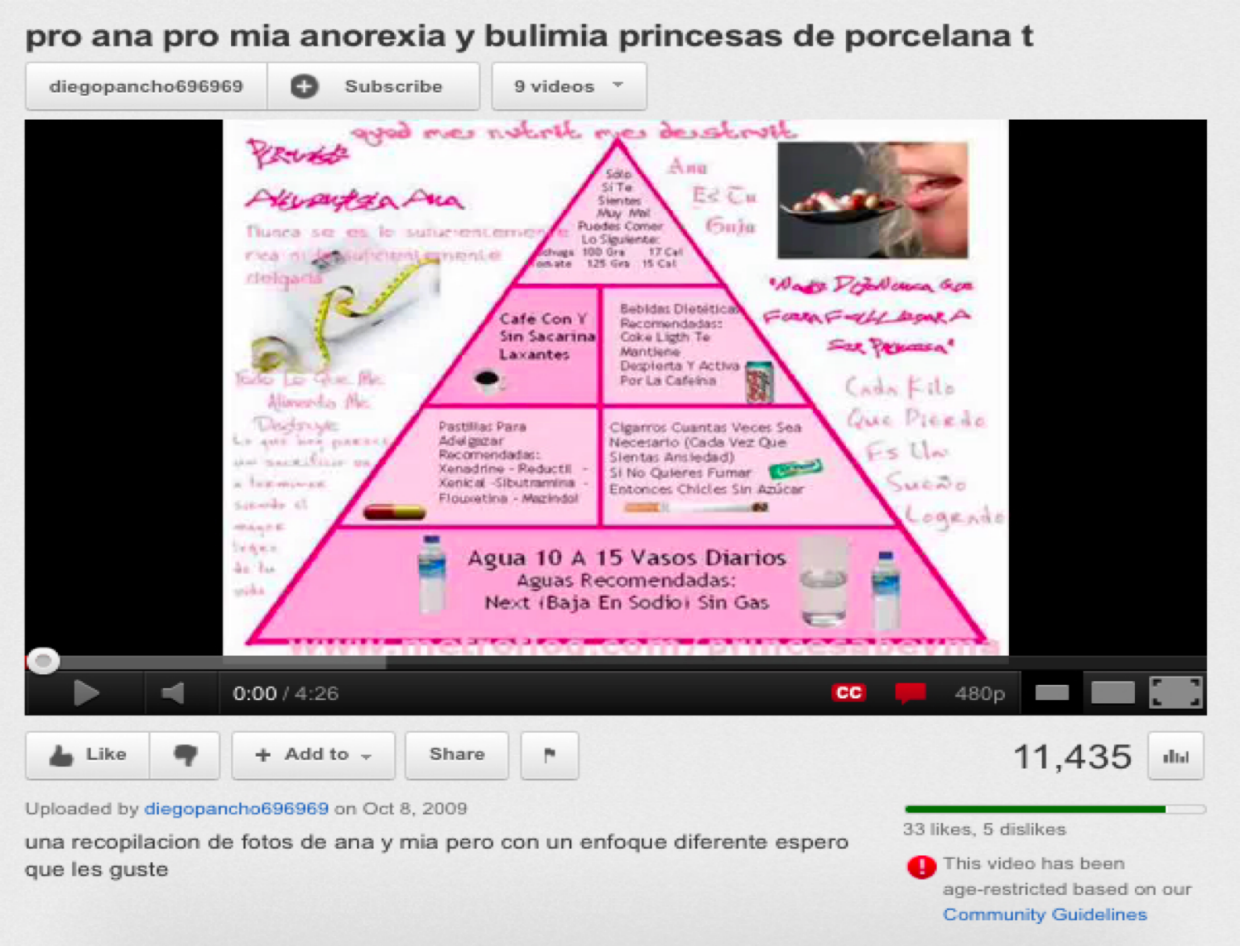
Screenshot of a Spanish video promoting anorexia through drugs and smoking.

**Figure 4:**
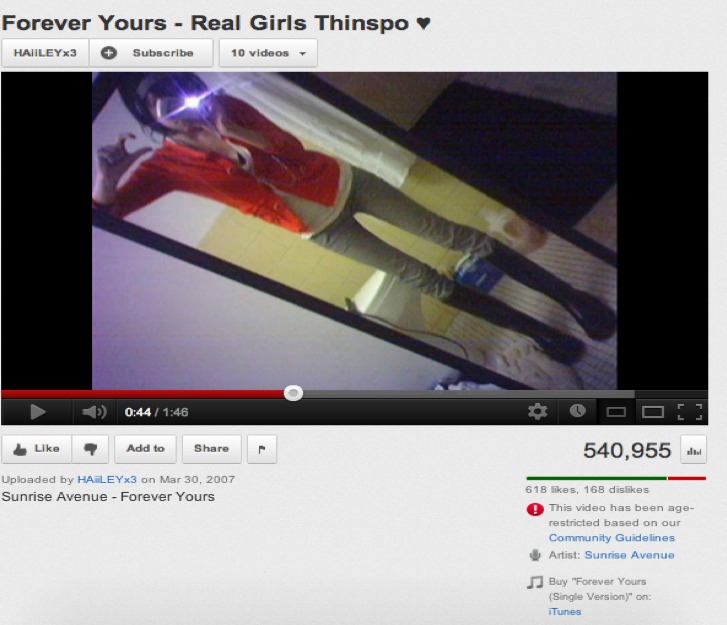
Screenshot of a video promoting anorexia featuring very thin models.

In most cases, the pro-anorexia videos featured photos of extremely thin models. These videos were explicitly used to inspire people to become very thin. It was also common for some of the videos to include quotations with tips and advice for losing weight. For example, Figure 3 is a screenshot of a video in Spanish with a thinspo nutritional pyramid with advice such as “Smoke as much as necessary, or eat sugar-free chewing gum, use drugs such as Xenadrine, Reductil, etc, to lose weight.” Nearly all the videos featured very thin female models (Figure 4), although we did encounter a few videos featuring very thin male models.

**Figure 5:**
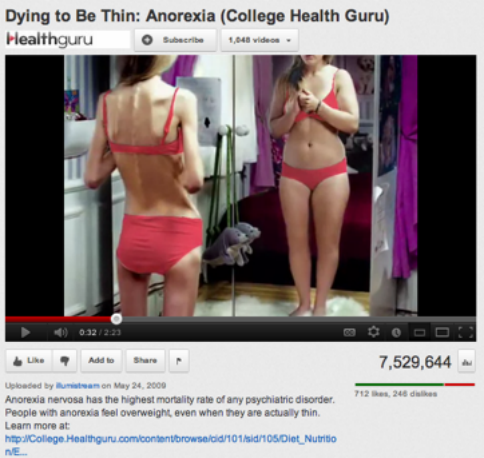
Screenshot of an informative video about anorexia.

Although we did not study the creators of these videos as a part of this study, we observed that a wide range of users provided the informative videos: individuals recovering from the disease, health organizations, news agencies, and students (see Figure 5). The most popular videos were produced by news agencies. Health authorities, such as the Centers for Disease Control and the National Health Service, also had videos about anorexia on YouTube.

In the others category, some videos were tagged with the keywords without any clear explanation. In other cases, the videos were from a music band called Anorexia.

**Figure 6:**
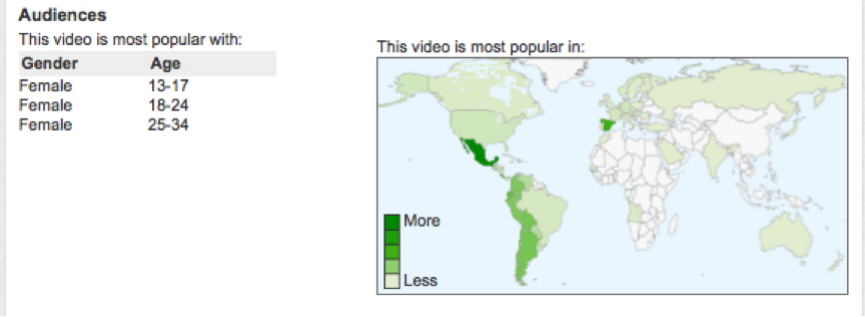
Demographics of the pro-anorexic video “Princesas de Porcelana” (Spanish).

To understand the demographic characteristics of the pro-anorexia community, we analyzed the demographic information available in the 15 videos from the pro-anorexia group (see Figure 6). A total of 15 pro-anorexia videos included demographic information, of which 80% (n=12) had minors (13-17 years) in a top-3 age group as viewers, with one-third (n=5) of the videos not having age restrictions. This implies that some videos were very popular among minors before being flagged as inappropriate for minors. Any registered user can flag videos as inappropriate, and then YouTube decides on the deletion or age restrictions for the videos based on flags from the community of users.

**Table 2:** Assessment of the 20 most-viewed anorexia-related videos on YouTube.

Variable		Video type				OR^a^ (95% CI)	*P* value
		Pro-anorexia		Informative			
		n	%	n	%		
Total views		9.51 million	100	51.62 million	100		
Favorite		24,462	0.26	39,424	0.08	3.37 (3.32-3.43)	<.001
**Total Responses** ^b^		15,209	0.16	45,486	0.09	1.82 (1.78-1.85)	<.001
	Likes	12,560	82.58^c^	40,332	88.67^c^	0.61 (0.58-0.64)	<.001
	Dislikes	2649	17.42^c^	5154	11.33^c^	1.65 (1.57-1.74)	<.001

^a^ OR: odds ratio (informative group is reference).

^b^ Response: videos were rated with like or dislike.

^c^The percentages of likes and dislikes were calculated by using total responses as denominator.

## Discussion

Video content on YouTube has been analyzed in previous studies as a source of information about the human papillomavirus vaccine, rheumatoid arthritis, influenza A (H1N1) virus, kidney stones, and immunizations [17-24]. To our knowledge, this is the first study to categorize and quantify anorexia-related videos on YouTube. We found a high prevalence of pro-anorexia videos on YouTube; nearly one-third of all anorexia-related videos promoted anorexia as a lifestyle (see Table 1). In both subsets of videos, the 110 most-viewed and the 30 randomly selected videos, the percentage of pro-anorexia videos was approximately 30% (see Table 1). These percentages are similar to studies analyzing YouTube videos on other health-related topics, such as rheumatoid arthritis and immunization [17,24]. If we extrapolate the percentage of pro-anorexia videos from our dataset to all the anorexia videos we extracted (n=7583), we can estimate that there are more than 2222 pro-anorexia videos on YouTube.

In this study, we found that the most-viewed informative videos were produced by news agencies. This could be because news agencies, such as CBS News, have a wide viewership and therefore any video uploaded by them will get many viewers. In some cases, the informative videos from news agencies reported cases of celebrities affected by the disease. The popularity of videos created by health authorities was relatively low compared with news agencies and some personal videos. Studying why videos from health authorities were not so popular did not fall within the scope of our study. However, we did observe that certain topics triggered many views (ie, fashion and celebrities), and these topics were not normally featured in health authority videos.

When we analyzed the characteristics of the most popular videos, we found that the pro-anorexia videos were favored 3 times more by viewers than the informative videos. The response rate was estimated from the number of viewers who clicked on the like/dislike icon over total views. The number of comments for pro-anorexia videos was twice that for informative videos. A study has reported that the interaction among the pro-anorexia community is very intense [39]. One of the reasons for the lower response to informative videos could be that some health authorities opt to block comments on their videos. Greater popularity, in terms of likes and comments, will increase the visibility of the video because search and recommender algorithms within YouTube promote highly linked and commented videos [40]. We observed differences in the use of textual descriptions of the videos; for example, many pro-anorexia videos were described as having tips for weight loss that may attract a wider audience than just pro-anorexia members. We encourage health authorities to study the content dissemination strategies used by the pro-anorexia users to design their own dissemination strategies for informative content. In addition, health authorities should see YouTube as an online community and engage with it to increase their popularity. Among other things, we recommend they involve other health authorities or research institutions and engage with the viewership via comments when possible.

Approximately 82.6% of pro-anorexia video raters liked the misleading information. In contrast, 11.3% of informative video raters disliked the informative content (see Table 2). We assume this is because misleading videos are made attractive with pictures of celebrities and models and fashionable music [35], whereas informative videos are often just simple lectures that are less visually appealing. This reflects the fact that even though there are a significant number of informative videos (55%), they are less favored than the pro-anorexia ones. Therefore, more effort is required to promote the visual appeal of informative videos. Merely increasing the number of informative videos does not necessarily correlate with the number of views.

We observed characteristics through the most-viewed pro-anorexia videos congruent with the review conducted by Rouleau and von Ranson [32] on the risk of pro-anorexia webs. First, the videos provide support such as emotional reinforcements via music, photos (see Figure 4), and quotations. The reinforcement of disordered eating is also common on the videos with the sharing of pro-anorexia tips (see Figure 3) and help seeking is discouraged by denying the disease exists (see Figure 1). Videos can be heavily pro-anorexia; in a few minutes and with an appealing format, they can combine all the risks identified by Rouleau and von Ranson [32]. Harshbarger et al [31] concluded in their study that the “tips and tricks” sections of pro-anorexia websites posed the most serious medical threat because the most frequent theme was dieting and calorie restriction. This is also true of the pro-anorexia videos on YouTube. As in many pro-anorexia websites [31], some videos recommend smoking, drinking a lot of water to avoid eating, and the use of laxatives and weight loss drugs (see Figure 3). A simple search on YouTube for “water fasting” retrieves more than 8000 videos. In addition to pro-anorexia and pro-eating disorder websites, music videos featuring thin models have also been shown to be an influential form of mass media for adolescents [38]. Although viewers pay less attention to these videos, exposure to them has led to increased body dissatisfaction [38]. Similarly, this study also found that the most popular pro-anorexia videos were music videos featuring thin models.

The flagging of videos as inappropriate to minors was shown to be of limited use. In fact, minors (age 13-17 years) were found to be the top viewers. The most logical explanation for minor viewers of flagged videos is that the minors were actively watching them before they were flagged. Another problem with flagging is its reliance on the data provided by users, which can be inaccurate [41]. Pro-anorexia content on social media can be particularly dangerous for minors because they may come across pro-anorexia content while searching for related topics, such as healthy diets. Therefore, on the one hand, awareness needs to be raised among teenagers about their perception of beauty and healthy lifestyles; on the other hand, future research needs to focus on the development of search algorithms to promote informative content and prevent harmful content from being accessible.

There is a common misconception that the accuracy of online information is directly related to the number of hits or views. In other words, the more hits or incoming links, the more relevant the information is in search algorithms such as PageRank [42]. The same applies to videos; more views mean the content is popular and, therefore, more accurate and relevant. However, this is not true. There are thousands of health-related videos promoting misleading information that garner millions of views. Studies reporting the characteristics of misleading information and the community that generates such content could be used to create more robust search engines to make it easier to find trustworthy content while filtering out misleading information. For example, Fernandez-Luque et al [6] explored the use of social network analysis to find relevant diabetes content on YouTube. In this study, we found (as shown in Table 2) that popularity favored misleading information. Therefore, a robust search engine will need to take into account other parameters, such as trust-based algorithms based on social network analysis or natural language processing (NLP) techniques, and not merely popularity. For example, NLP has demonstrated its ability to extract information and relations from texts [43]. A classification of the content according to its informative nature could be performed by analyzing the metadata of extracted videos, as proposed by Himmel et al [44] on health forums.

### Study Limitations

This study was limited to the content analysis of videos retrieved on October 10, 2011, from YouTube; it was not replicated in other video platforms, such as Vimeo. Therefore, the external validity of the data is limited and may not be generalized to overall health-related videos available on the Internet. The classification performed by physicians was subjective, although definitions for each category were provided. We did not use thinspo as a separate criterion. However, during our group discussions we agreed that videos about the inspiration to become thin (thinspo) should be rated as videos promoting anorexia. In addition, referring to pro-anorexia videos as misleading is a simplification because they can be informative, but still harmful from a health point of view.

The data used for this study was from YouTube and was anonymous. For example, it is virtually impossible to be certain about the identity of those responsible for creating or uploading the videos because individual users may have (illegally) uploaded videos using false email identification. In addition, the age of users cannot be confirmed because minors can fake their age to gain access to restricted content.

We were interested in the videos with the maximum number of viewers and not the number of videos per se; therefore, we selected the top 40 videos, not all 140 videos. These 40 videos had a total of 61.13 million views, which is a large enough sample size to understand the characteristic features of the viewers. Furthermore, search engine optimization experts suggest that 95% of search engine users do not go beyond 2 pages of search results [45]. The interrater agreement in the study was only moderate, primarily because of the complexity of classifying videos that combined music, photos, and text; in 10 cases, the text was not congruent with the photos. We conducted an additional review process to resolve discrepancies pending from the first review.

### Conclusion

In this study, we found and quantified the presence of content promoting anorexia on YouTube, the most popular video site. Pro-anorexia videos are less common than informative videos; however, pro-anorexia content is highly favored and rated by users. Another problem identified in our study is the popularity of pro-anorexia videos among young viewers. Health authorities generating health videos on anorexia should be aware of the presence of the pro-anorexia communities and the strategies they use to reach a wider audience, such as featuring models and celebrities.

With the rapid development of information and communication technologies (ICT), digital information is becoming widely available on mobile devices. Social networking websites are acting as catalysts for the dissemination of information. To rephrase Alvin Toffler, the illiterate in this ICT era will not be those who cannot read and write, but those who cannot distinguish between trustworthy and misleading information available online [46]. Most viewers of videos with misleading information are minors; therefore, children need to be taught how to discern between trustworthy and misleading information at school. Health authorities should involve models and celebrities to help them promote health-related information. Researchers should not confine themselves to journals (research communities), but share their research findings on social network sites [47,48]. Laypeople prefer to search for information on the social platforms rather than in scientific journals. We recommend active participation from health institutions and individual researchers to promote informative videos. Active participation also includes flagging (or denouncing) misleading videos.

In addition, more research is required to identify misleading content automatically by using filtering algorithms based on the different characteristics of pro-anorexia and informative videos. A recent study of pictures promoting anorexia in a photo-sharing community [39] found that social and textual clues could be used to automatically identify pro-anorexia pictures. These approaches could be used to filter pro-anorexia content before it is published.

## Multimedia Appendix 1

Example of a pro-anorexia video “Thinspo (Syntax Error - Träumen im Gras)” by MelinaAnaJolie, from http://www.youtube.com/watch?v=QD3nLj58j2Q (http://www.webcitation.org/6EP3xdcPt), reproduced under Creative Commons Attribution License.

## Multimedia Appendix 2

Example of an informative video “10 Things I want Parents to Know About Anorexia” by 101daisysaisy, from http://www.youtube.com/watch?v=j8txQmvbIN4 (http://www.webcitation.org/6EP4COCGC), reproduced under Creative Commons Attribution License.

## Abbreviations

API: application programming interface
ICT: information and communication technologies
NLP: natural language processing
OR: odds ratio
